# Migratory Restlessness and the Role of Androgen for Increasing Behavioral Drive in the Spawning Migration of the Japanese eel

**DOI:** 10.1038/srep17430

**Published:** 2015-11-30

**Authors:** Ryusuke Sudo, Katsumi Tsukamoto

**Affiliations:** 1 Aquaculture Systems Division, National Research Institute of Aquaculture, Fisheries Research Agency, 422-1 Nakatsuhamaura Minami-Ise, Mie 516-0193, Japan; 2 Department of Marine Science and Resources, College of Bioresource Science, Nihon University, 1866 Kameino, Fujisawa, Kanagawa, 252-0880 Japan

## Abstract

Migratory restlessness refers to a type of locomotor activity observed just before the onset of a migration. This behavior is primarily known in birds, where it is considered to be an indicator of the urge for migration. In contrast, little is known about migratory restlessness in fishes. To confirm migratory restlessness in a fish, we measured the locomotor activity of the Japanese eel, *Anguilla japonica* during its migration season. Migratory-phase silver eels showed higher locomotor activity in aquaria than yellow eels at the non-migratiory growth-phase. Silver eels stayed outside of their shelters for longer durations in dark periods than yellow eels and were active even in light periods when yellow eels were inactive in the shelters. Silver eels had higher levels of the androgen hormone 11-ketotestosterone at the end of experiment than yellow eels. Administration of 11-ketotesosterone to yellow eels induced higher levels of locomotor activity than that observed in non-treated controls. These findings suggest that anguillid eels exhibit migratory restlessness just before their spawning migration and that 11-ketotestosterone may be involved in the onset of this behavior.

Migratory restlessness (also known as *Zugunruhe*) is the seasonally occurring behavior of caged migratory birds that is expressed by high locomotor activity during migration seasons and is considered to be an indicator of the urge for migration^1^. Studies of migratory restlessness have greatly contributed to the understanding of migration in birds, including the endogenous basis of migratory behavior and the direction of the migration route^1^,^2^. Some kinds of fish also exhibit dynamic migrations, and this is especially true for diadromous fishes that move between freshwater and the sea. Possibly, some of these fishes also exhibit migratory restlessness just before their migrations. However, compared to birds, little attention has been focused on this aspect of the migratory behavior of fishes.

Catadromous anguillid eels are one of the most well-known and widely distributed types of diadromous fishes along with salmon. They are famous for their long-distance spawning migration of thousands of kilometers, and have a complex life cycle. After a long period of growth in inland waters or estuaries, yellow eels undergo marked morphological and physiological changes as they transform into the silver eel phase, which commence to migrate back to the open ocean to spawn^3^,^4^. This change is termed ‘silvering’ and is a preparatory adaptation for the spawning migration in the oceans. During silvering, some behavioral differences, such as a rheotaxis^5^ and locomotor activity, also occur. For example, a sudden drop in temperature caused an increased locomotor activity in silver eels, but not in yellow eels^6^. In addition, a recent study demonstrated that silver eels showed higher locomotor activity than yellow eels in outdoor tanks, which reflected the river water conditions^7^. That study also revealed that increased activity of silver eels was stimulated by a rise in turbidity, which is thought to be one of the triggering factors for their spawning migration^7^. These findings suggest that silver eels exhibit migratory restlessness before starting their spawning migration. Therefore, eels may provide a useful model for studying migratory restlessness in fishes.

In birds, migratory restlessness is used as an indicator of internal drive for migration. Thus, their activity is measured in bird cages that are isolated from meteorological factors. For confirming whether migratory restlessness in eels is reflecting internal drive for spawning migration or not, we have to demonstrate that higher activity only occurs in migratory silver eels that are held in tanks isolated from meteorological factors.

In diadromous fishes, endocrine regulation has been thought to play a major role in motivating migratory behavior^8^, an androgens are believed to contribute to the onset of the spawning migration in eels^9^. For example, increases in the levels of testosterone and 11-ketotestosterone (11-KT) were reported during silvering in both female and male eels^10^,^11^,^12^,^13^. Androgen treatment of eels resulted in silvering-related changes, such as increases in eye diameter and skin thickness, and degeneration of the digestive tract^12^,^14^,^15^. Recently, 11-KT treatment was shown to induce a higher frequency of movements between freshwater and seawater, which may be related to migratory restlessness^16^. Thus, the objectives of this study were to confirm the presence of migratory restlessness in eels and to examine the role of androgens in the drive to start the spawning migration in eels by measuring the locomotor activity of 11-KT treated eels.

## Results

**Figure 1:**
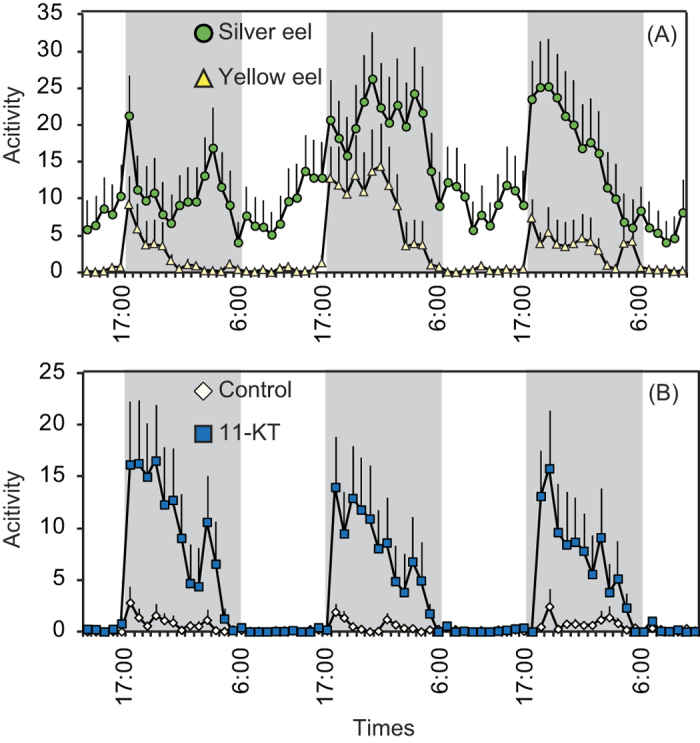
Locomotor activity of Japanese eels. Activity (mean ± SE) was measured over 72 h for (**A**) non-migrating (yellow) and migrating (silver) eels, and (**B**) 11-KT treated yellow eels and non-treated control yellow eels. Gray shading indicates dark-periods.

**Figure 2:**
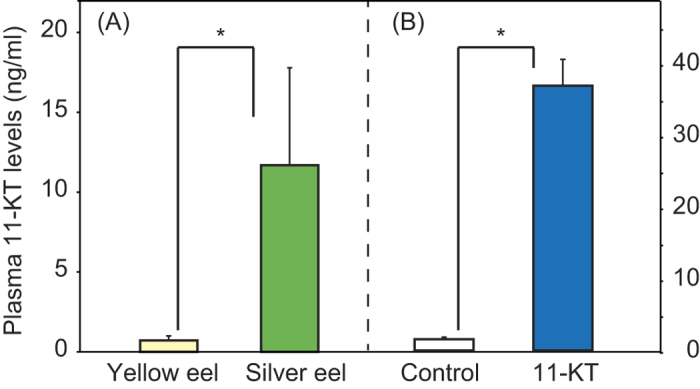
Androgen levels in yellow eels and silver eels (**A**), 11-KT treated eels and control eels (**B**).Plasma 11-KT concentrations (mean ± SE) were measured in eels at the end of the 72 h period of observation. Asterisks indicate significant differences between two groups.

In both experiments, all eels exhibited generally higher levels of locomotor activity during the dark periods than during the light periods (Fig. 1). Yellow eels were most active in the early nighttime and showed almost no activity during the day (Fig. 1A). Silver eels exhibited higher locomotor activity than yellow eels throughout the experimental period. At the end of the experiment, plasma 11-KT concentrations were significantly higher in silver eels than in yellow eels (U-test, p < 0.001) (Fig. 2). In the androgen administration experiment, 11-KT treated eels showed high locomotor activity immediately after the lights were switched off. Their activity generally declined until the morning, when most activity had ceased. In contrast, non-treated control eels showed variable and much lower activity during the night and very little activity during the day (Fig. 1B). Plasma 11-KT concentrations were significantly higher in 11-KT treated eels than in control eels whose 11-KT levels were similar to that of yellow eels (U-test, p < 0.001).

**Figure 3:**
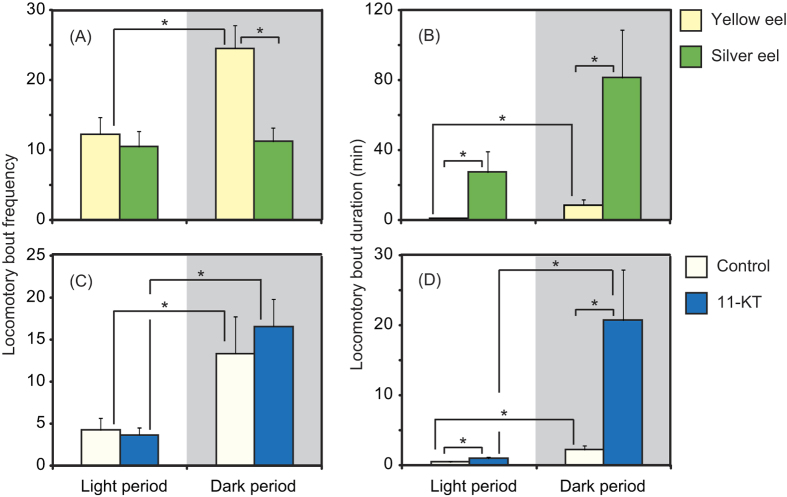
Movements of yellow and silver eels outside their shelters showing locomotory bout frequency (**A**,**C**) and locomotory bout duration (**B**,**D**) in the two experiments. Asterisks indicate significant differences between the indicated groups of eels.

Besides the difference between silver eels and yellow eels in their absolute levels of activity as shown in Fig. 1a, there were differences with respect to the other types of activity measurements. During the dark period, yellow eels moved in and out of the shelters more frequently than did the silver eels. Consequently, the locomotory bout frequencies were statistically higher in yellow eels (U-test, p < 0.05; Fig. 3A), but they moved in and out of the shelters much less frequently during the light period than during the dark period. Although, during the light period, there was no statistical difference between yellow and silver eels in locomotory bout frequency, yellow eels remained outside the shelters for statistically less time on each occasion (U-tests, p < 0.05). The locomotory bout duration in silver eels was significantly higher than in yellow eels during the dark period (U-test, p < 0.05) (Fig. 3B). The locomotory bout frequency and duration differed between light and dark periods in yellow eels (U-test, p < 0.05), whereas they did not in silver eels (Fig. 3A,B).

In the 11-KT administration experiment, there were no significant differences between treated and non-treated eels in locomotory bout frequency within light or dark periods but, in both types of eels the values were significantly higher in dark periods compared with light periods (U-test, p < 0.05; Fig. 3C). In contrast the treated eels showed significantly higher values than non-treated eels for locomotory bout duration within both the light and dark periods (U-test, p < 0.05). Both treated and non-treated eels exhibited statistical differences in locomotory bout frequency and duration between the light and dark periods (U-test, p < 0.05; Fig. 3D).

## Discussion

In the present study, a number of differences between silver and yellow eels were observed in their activity levels and behaviors. Silver eels were captured in Hamana Lake during their downstream migration season, presumably just before they were going to enter the ocean for their long migration to their spawning area, and they showed much higher activity levels than non-migrating yellow eels from the lake. Silver eels were active during both day and night and tended to stay outside their shelters for longer periods. In comparison, yellow eels showed little activity during the light period and moved in and out of their shelters more frequently in dark periods but did not remain outside the shelters as long as silver eels. These findings are not surprising because silver eels might be expected to be more motivated to escape from confinement in a small spaces to resume their migrations. Indeed, silver eels have been observed to leave the water to escape if necessary^4^. The elevated day time activity of silver eels compared with yellow eels is also significant, because anguillid eels generally show a clear negative phototaxis by hiding during the day, and usually emerging to feed only at night^17^. The fact that the silver eels also remained outside of their shelters for longer periods than yellow eels during the day, may indicate an urge for movement, consistent with a motivation to resume their spawning migration.

The higher 11-KT levels in silver eels than in yellow eels at the end of the experiment is consistent with previous studies, which have found that this hormone is associated with the silvering process^10^,^11^,^12^,^13^. The 11-KT administration experiment in the present study showed that in addition to being linked to silvering in eels, 11-KT induced increased the locomotor activity of yellow eels in dark periods. The differences in activity between 11-KT treated eels and control eels were similar to the differences between yellow eels and silver eels in the previous experiment, although the activity levels were lower in the hormone treated eels than in non-hormone treated silver eels. The 11-KT treated eels were similar to the silver eels in the first experiment with respect to the other behavioral measurements though; because their outside shelter durations were much greater than in untreated eels, while their numbers of outside shelter movements were similar. This clearly indicates that 11-KT administration increased the motivation for the treated eels to elevate their locomotor activity and to remain outside their shelters for longer than untreated yellow eels. This occurred even though the treated eels were not yet undergoing obvious reproductive maturation (Table 1).

The higher locomotor activity of silver eels and the increased locomotor activity in yellow eels triggered by 11-KT administration in our study resemble migratory restlessness in birds in some respects. Eels leave their growth habitats during their downstream migration season and commence a long oceanic migration at the silver stage, whereas yellow eels are more sedentary while usually remaining in well-defined home ranges throughout the year during their resident growth phase^18^. In migratory birds, migratory restlessness occurs only in the migratory season of each particular bird^1^,^2^. Another similarity is that the apparent migratory restlessness in both birds and eels occurs in the absence of direct meteorological cues. The activity of migratory birds has been measured in enclosed laboratory conditions isolated from meteorological factors, indicating that their migratory restlessness must be derived from internal factors. In this study, locomotor activity was also measured in enclosed recirculating aquaria in a laboratory to eliminate most meteorological factors that could potentially affect the locomotor activity of eels^7^. A third similarity is in the characteristics of the daily patterns of higher activity in birds and eels. In silver eels, their negative phototaxis was reduced during the day compared with yellow eels. Birds exhibiting migratory restlessness are active nocturnally, while they normally are only active during daylight. These changes in diurnal activity patterns may be a characteristic of migratory restlessness, although the change is opposite between birds and eels. These similarities suggest that the higher activity observed in the silver eel phase of anguillid eels can probably be considered as a form of migratory restlessness. There is also some evidence that this motivation for migration may be increased during the low-light periods of the lunar cycle^19^,^20^. In this study, the observation period was limited and thus we could not clarify the effect of photoperiod, which triggers migratory restlessness in birds^1^,^2^. For understanding of the role of photoperiod in eel migration, seasonal behavioral observations are needed in the future.

To further investigate the higher locomotor activity of silver eels that appears to be a form of migratory restlessness, we focused on the roles of androgen. From hormone measurements, it is clear that the gonadotropic axis is activated during silvering^9^,^21^. Among the various reproductive hormones, androgens, especially 11-KT are markedly increased during silvering^10^,^11^,^12^,^13^. In addition, it was recently reported that 11-KT treatment induced a higher frequency of movements between freshwater and seawater, which may indicate restlessness^16^. Thus, we carried out the 11-KT treatment to determine whether androgen is involved in inducing migratory restlessness in eels. The results of the experiments clearly demonstrated that 11-KT administration increased nighttime locomotor activity in yellow eels. Because the difference between hormone treated eels and control eels was quite similar to the difference between yellow eels with low measured 11-KT levels and silver eels with high 11-KT levels, it appears that 11-KT may play an active role in inducing migratory restlessness in eels.

In European eels, androgen was found to stimulate brain dopaminergic systems, which may have an influence on these types of behavior^22^. This observation together with those of the present study indicate that 11-KT is involved in silvering and also in migratory behavior at the onset of the spawning migration. Recently, we revealed that gradual water temperature decrease (from 25 °C to 15 °C) that simulate the temperature changes during the autumn migratory season, induced elevation of 11-KT^23^. This suggests a possible scenario of downstream migration being triggered in silver eels, by decreasing water temperature in the autumn, which induces the release of 11-KT, that then elevates the migratory drive. This type of model has been proposed for diadromous fishes by Tsukamoto *et al.* 2009^24^.

In conclusion, we confirmed that silver eels exhibited higher locomotor activity and a reduction of negative phototactic behavior during their spawning migration season compared with yellow eels. This stage-specific higher locomotor activity appears to be migratory restlessness and occurs in enclosed aquaria, which were isolated from meteorological factors. Therefore, this migratory restlessness may be reflecting the internal motivation to start spawning migration of eels, as migratory restlessness does in birds. In addition, higher activity levels were induced in non-migrating yellow eels by 11-KT administration, which suggests this hormone may be directly involved in the elevation of the drive for spawning migration in silver eels. This is new direct evidence for the relationship between migratory behavior and hormones in anguillid eels.

## Materials and Methods

### Ethics

All experiments including fish handling and processing were conducted in accordance with the “Principles of morality in animal experiments (ethics protocol 4-3-3 and 4-3-4)” of The University of Tokyo, and were approved by The University of Tokyo.

### Collection of eels

This study used female Japanese eels collected in Hamana Lake of central Japan. In 2008, eels were collected using fyke nets and eel pots by local fisherman on 1, 9, 16, 23 November and 1 December. Six to ten eels were caught in each collection period and were classified as yellow eels or silver eels, according to a silvering index^25^. Both yellow and silver eels were directly transferred into experimental aquaria at same time for the behavioral observations of each 5 trials as described below. In 2011, 32 yellow eels were caught in September and October and transferred to the laboratory and maintained in two tanks (500 L) containing brackish water (30 psu, 18 °C) until they were used in the 5 trials of the androgen administration experiment. Eels were held in a natural photoperiod before being tested. For this experiment, six or eight eels were randomly selected from the rearing tanks and separated into the two groups for each trial.

### Administration of 11-KT

Sylastic tubes, which have been used previously for chronic delivery of sex steroids, were used for 11-KT administration to 16 yellow eels^14^,^15^. The 11-KT was dissolved in ethanol and castor oil (Sigma-Aldrich) mixed in a 1:9 ratio, and encapsulated in sylastic tubes at a dose level of 0.1 mg 11-KT per kg of body weight. For the control group, sylastic tubes containing only ethanol and castor oil were used. All eels were anesthetized with clove oil (0.2%), and the sylastic tube were inserted in their abdominal cavities. The weight of the sylastic tubes for administration of 11-KT were approximately 1.5 g, and the maximum percent weight of the sylastic tubes relative to the BW of the eels was 0.7%. Thus they were not a burden on the bodies of the eels.

### Behavioral observations

Behavioral observations were performed using 10 glass aquaria (90 cm long, 45 cm wide, 40 cm high, with 30 cm water depth), with each aquarium containing one eel. Re-circulating brackish water (30 psu) continuously flowed through each aquarium system and was filtered with a mixture of charcoal and silicon sand wrapped with wool fiber. We set up two contrasting light intensities that were intended to represent light or dark periods. Lighting was provided by a fluorescent lamp for each aquarium placed 15 cm above the water surface (50 lux at the bottom of the aquarium without water) from 6:00 to 17:00 (the light period) and by two infrared lamps (IR100; Toshiba) 3.0 m from the side of each aquarium (7 lux) from 17:00 to 6:00 (the dark period). The water temperature was maintained at 18 °C during the experiment, because in Hamana Lake, silver eels tend to start to migrate at 15–20 °C. In the aquaria, a single polyvinyl tube (5 cm diameter, 60 cm long) was provided as shelter for the eels.

In 2008, yellow and silver eels were transferred to experimental aquaria on the day of collection and were allowed to acclimatize for three days before starting the experiment. In 2012, yellow eels were transferred to stock tanks where they were reared until 11-KT administration. After 11-KT administration, they were transferred to aquaria and acclimated for one day before starting the video recording. After acclimation, continuous videotaping from the front window of the aquaria (video camera: Ikegami CCD camera ICD-878; Recorder: Sharp Digital Hi-Vision Recorder and DV-HRD 200; monitor: Victor, Casio Cordless Vision XF-800) was performed for three days in both experiments. We carried out five trials in 2008 (1–4, 9–13, 16–19, 23–26 Nov, and 1–7 Dec) and five trials in 2011 and 2012 (31 Dec to 3 Jan, 8–11 Jan, 2–5, 9–12, 24–27 Nov) under laboratory conditions. Each trial lasted for 72 h for both the yellow and silver eel activity experiment and the 11-KT administration experiment.

It has been observed that eels exhibited an increase of locomotor activity with decreasing shelter availability^26^. This characteristic of movements in relation to hiding in shelters has been used for measuring locomotor activity of eels^6^,^7^,^27^. Therefore, this study examined the number of movements out of the shelters in three different ways to evaluate the activity levels of the eels in the two experiments. Using the video recordings, we measured the time between departure from a shelter and return to a shelter, and refer to this as a locomotory bout, and defined locomotor activity as bout duration per hour. We also measured locomotory bout frequency and locomotory bout duration, which is defined as the time spent during each locomotory bout.

### Morphological measurement and blood sampling

After the behavioral observations, all eels from both experiments, were anesthetized with 0.08% 2-phenoxyethanol, and the following external measurements were made on each eels: total length (TL), body weight (BW), and horizontal and vertical eye diameter (Dh and Dv). Blood samples were taken from the bulbus arteriosus using heparinized syringes. After centrifuging at 3300 g for 20 min at 4 °C, plasma was collected and stored at –20 °C until being used for steroid measurement. After the collection of the blood samples, the ovaries and gut were dissected and weighed. Eye index (EI), and gonado-somatic index (GSI) were calculated using the following formulae: EI = [{(Dh + Dv)^2^/4} × *π*]/TL, GSI = gonad weight/BW × 10^2^, GI = digestive tract weight/BW × 10^2^. The morphological parameters of eels used in this study are summarized in Table 1.

### Measurement of 11-KT

The concentrations of 11-KT were measured in plasma samples by time-resolved fluoroimmunoassay (TR-FIA) according to the method of Yamada *et al.* 1997^28^. Blood samples for TR-FIA were prepared by ether extraction, and the extracts were reconstituted in assay buffer. 11-KT conjugate was prepared by the methods of Asahina *et al.* 1995^29^. The steroid-BSA conjugate was immobilized in the wells of microtiter plates (4 °C, overnight). After three washes with 0.9% saline, the wells were blocked with 0.1% BSA, followed by three washes for immunoassays. Twenty-five microliters of standard or extracted serum samples and 75 μl antisteroid sera for 11-KT (Cosmo Bio Co., Ltd., Tokyo, Japan) were dispensed to each well. After the immunoreactions (4 °C, overnight) and three washes, europium-labeled anti-rabbit immunoglobulin G (IgG) goat IgG (Eu-IgG, PE Applied Biosystems) was added to the wells, and the plates were shaken for 1 h at room temperature. Eu was dissociated from the steroid complex using a primary antibody and Eu-IgG by addition of an enhancement solution. The intensity of Eu was measured by a using multilabel counter (1420 ARVO-D, PE Applied Biosystems).

### Data analysis

We plotted time-series graphs of locomotor activity of each groups for the entire 72 h of video recording of both experiments. Comparisons of 11-KT concentrations, bout frequency and bout duration were analyzed using the Mann-Whitney U-test. All statistical analyses were performed using Excel 2013 (Microsoft, USA) with the add-in software Excel stat (SSRI, Japan).

## Additional Information

**How to cite this article**: Sudo, R. and Tsukamoto, K. Migratory Restlessness and the Role of Androgen for Increasing Behavioral Drive in the Spawning Migration of the Japanese eel. *Sci. Rep.* **5**, 17430; doi: 10.1038/srep17430 (2015).

## Figures and Tables

**Table 1:** Morphological parameters and 11-KT concentrations of eels used in this study.

Year of study	2008		2011–2012	
	Yellow eel	Silver eel	Control	11 KT treatment
n	18	21	16	16
TL	58.5 ± 7.5	71.2 ± 6.5	70.7 ± 8.2	71.7 ± 8.2
BW	310 ± 156	622 ± 176	573 ± 230	594 ± 230
GSI	0.59 ± 0.13	2.48 ± 0.79	1.77 ± 0.47	1.99 ± 0.47
EI	3.87 ± 0.83	6.64 ± 1.17	6.69 ± 0.99	6.88 ± 0.99
GI	1.74 ± 0.38	0.54 ± 0.32	0.73 ± 0.32	0.69 ± 0.32
